# DBCSMOTE: a clustering-based oversampling technique for data-imbalanced warfarin dose prediction

**DOI:** 10.1186/s12920-020-00781-2

**Published:** 2020-10-22

**Authors:** Yanyun Tao, Yuzhen Zhang, Bin Jiang

**Affiliations:** 1 Intelligent transportation and cognitive computing laboratory, Soochow university, Shizi Street 1, Suzhou, 215005 China; 2 the Cardiovascular Department, the First Affiliated Hospital of Soochow University, Shizi Street 100, Suzhou, 215005 China

**Keywords:** Warfarin dose prediction, Data imbalance, Oversampling, Density clustering, Predictive models

## Abstract

**Background:**

Vitamin K antagonist (warfarin) is the most classical and widely used oral anticoagulant with assuring anticoagulant effect, wide clinical indications and low price. Warfarin dosage requirements of different patients vary largely. For warfarin daily dosage prediction, the data imbalance in dataset leads to inaccurate prediction on the patients of rare genotype, who usually have large stable dosage requirement. To balance the dataset of patients treated with warfarin and improve the predictive accuracy, an appropriate partition of majority and minority groups, together with an oversampling method, is required.

**Method:**

To solve the data-imbalance problem mentioned above, we developed a clustering-based oversampling technique denoted as DBCSMOTE, which combines density-based spatial clustering of application with noise (DBCSCAN) and synthetic minority oversampling technique (SMOTE). DBCSMOTE automatically finds the minority groups by acquiring the association between samples in terms of the clinical features/genotypes and the warfarin dosage, and creates an extended dataset by adding the new synthetic samples of majority and minority groups. Meanwhile, two ensemble models, boosted regression tree (BRT) and random forest (RF), which are built on the extended dataset generateed by DBCSMOTE, accomplish the task of warfarin daily dosage prediction.

**Results:**

DBCSMOTE and the comparison methods were tested on the datasets derived from our Hospital and International Warfarin Pharmacogenetics Consortium (IWPC). As the results, DBCSMOTE-BRT obtained the highest *R*-squared (*R*^2^) of 0.424 and the smallest mean squared error (mse) of 1.08. In terms of the percentage of patients whose predicted dose of warfarin is within 20% of the actual stable therapeutic dose (20%-*p*), DBCSMOTE-BRT can achieve the largest value of 47.8% among predictive models. The more important thing is that DBCSMOTE saved about 68% computational time to achieve the same or better performance than the Evolutionary SMOTE, which was the best oversampling method in warfarin dose prediction by far. Meanwhile, in warfarin dose prediction, it is discovered that DBCSMOTE is more effective in  integrating BRT than RF  for warfarin dose prediction.

**Conclusion:**

Our finding is that the genotypes, CYP2C9 and VKORC1, no doubt contribute to the predictive accuracy. It was also discovered left atrium diameter, glutamic pyruvic transaminase and serum creatinine included in the model actually improved the predictive accuracy; When congestive heart failure, diabetes mellitus and valve replacement were absent in DBCSMOTE-BRT/RF, the predictive accuracy of DBCSMOTE-BRT/RF decreased. The oversampling ratio and number of minority clusters have a large impact on the effect of oversampling. According to our test, the predictive accuracy was high when the number of minority clusters was 6 ~ 8. The oversampling ratio for small minority clusters should be large (> 1.2) and for large minority clusters should be small (< 0.2). If the dataset becomes larger, the DBCSMOTE would be re-optimized and its BRT/RF model should be re-trained. DBCSMOTE-BRT/RF outperformed the current commonly-used tool called Warfarindosing. As compared to Evolutionary SMOTE-BRT and RF  models, DBCSMOTE-BRT and RF models take only a small computational time to achieve the same or higher performance in many cases. In terms of predictive accuracy, RF is not as good as BRT. However, RF still has a powerful ability in generating a highly accurate model as the dataset increases; the software “WarfarinSeer v2.0” is a test version, which packed DBCSMOTE-BRT/RF. It could be a convenient tool for clinical application in warfarin treatment.

## Background

Patients who have atrial fibrillation, valvular heart disease, thromboembolic disease, etc. need a long-term oral anticoagulant therapy. Vitamin K antagonist is the most classical and widely used oral anticoagulant with assuring anticoagulant effect, wide clinical indications and low price. Warfarin is the main Vitamin K antagonist comprehensively used in the clinic around the world. In recent years, although new oral anticoagulants (NOACs) are easy to use, they are relatively expensive, and their indications are relatively limited. Specific antagonists of NOACs are expensive and have not yet been marketed in China. NOACs efficacy can hardly be evaluated by routine tests or blood concentration tests. In contrast, although warfarin needs to monitor international normalized ratio (INR) [1–3] for stable dosage adjustment, its controllability and applicability of special symptoms make it the most cost-effective anticoagulant. Once the individual initial dosage can be accurately predicted, the number of dosage adjustments before stabilization can be reduced, anticoagulation effectiveness and safeness of warfarin can be improved, and the mortality of thromboembolism can be reduced. Individualized dose prediction of warfarin is a hot research topic in the field of anticoagulant individualized therapy in recent years. With the long-term accumulation of warfarin medical data, the volume and information integrity of the data are increasing, which provides a basis for the establishment of individual precise dose prediction model of warfarin by machine learning method.

Warfarin dosing should be precise for each patient. However, warfarin dosage requirements of different patients vary largely. For an individual patient, before the INR becoming stable within the therapeutic range and his or her stable dose being figured out, he or she has to endure quite a long time of numerously blood tests and dosage adjustments. And this process is mainly depended on doctor’s experience. To improve the predictive accuracy, many works have developed their warfarin dose predictive models [4–8] based on linear regression, such as the well-known predictive model of International Warfarin Pharmacogenetics Consortium (IWPC), Warfarindosing predictive tool, and Yu model, etc. [9–11]. Machine learning methods [12] such as boosted regression tree (BRT) [13], artificial neural networks (ANNs) [14, 15] and support vector regression (SVR) [16] can provide highly-accurate prediction in warfarin daily dose. BRT employed a gradient boosting to combine multiple binary regression trees [17]. We proved that BRT was able to present accurate prediction on warfarin dosage. Random forest (RF) integrated with an oversampling method has been applied in this area [18].

In 2018, Saffian et al. analyzed 22 predictive models for warfarin dose prediction before 2018 [19], and concluded that these models underpredicted the patients who require actual dosage > 7 mg/day. They claimed that it is necessary to deeper understanding of coagulation mechanisms such that the performance of predictive models can be improved. In addition, they didn’t give the reason for inaccuracy of these models. As reported in Ref [20], the genotype contributes to 43% dose variability. According to the research [21], the patients with VKORC1 of GG and CYP2C9 of *3/*3 were the minority, and the others were the majority. The data imbalance leads to inaccurate prediction of models on the patients with rare genotype, who usually have large stable dosage requirement. These previous works haven’t taken the data imbalance into account.

To balance the data and improve the overall predictive accuracy, appropriate partition of majority and minorities followed an oversampling technique is needed. In our previous works [21, 22], the dataset is divided depending on the value of genotypes and important clinical variables. However, the partition that only involves a few variables may not accurately obtain minority, whose members are sparse samples or outliers. Synthetic minority oversampling technique (SMOTE) have been tried [21] and evolutionary SMOTE (ESMOTE) was further proposed to optimize oversampling parameters of for data balance [22]. However, both of them just focused on improving the oversampling quality of minority samples, not care about finding appropriate minority classes.

Warfarin dose prediction is a typical regression problem. Distinguished from a classification problem, the labels of warfarin dose prediction are continuous warfarin dosage (mg/daily). There is no label for dividing minority or majority classes. To accurately find minority classes, only unsupervised learning methods can be used. The *k*-mean algorithm was used to distinguish the majority and minority [23]. However, *k*-mean cannot accurately find outliers or boundary samples, which were probably the minority. In this study, we propose a clustering-based oversampling method, which integrates density-based spatial clustering of application with noise (DBSCAN) [24] and SMOTE [25], to improve the warfarin dosage prediction. For convenience, this method is denoted as DBCSMOTE. DBSCAN conducts density clustering to find minorities in the warfarin dose-effect dataset, and SMOTE creates new samples for the minority class. The performance of DBCSMOTE is evaluated by the dose prediction of a predictive model built on the training set extended by BDCSMOTE. The ensemble models, BRT and RF, are used to accomplish this task.

### Dataset

Our dataset comes from two sources. One source contains 357 patients, who underwent warfarin treatment in the Suzhou area, was provided by the Department of Cardiology in The First Hospital of Soochow University. The other source contains 235 Han Chinese patients treated with warfarin, got from IWPC database. So, the total number of samples in our dataset is 592. The dataset is divided into a training set of 394 samples, a validation set of 50 samples and a test set of 148 samples.

Table 1 lists 13 clinical variables and two genotype variables in warfarin treatment. Many clinical factors can affect the anticoagulation effect of warfarin, including age, height, weight, gender, amiodarone and target INR, which are widely acknowledged as important factors that affect warfarin dosing. Amiodarone is an antiarrhythmic medication used to treat ventricular tachycardia or ventricular fibrillation, which is more likely to be taken together with warfarin than the other drugs. It has been proven that the genotypes have a remarkable influence on warfarin dose-effect. Congestive heart failure (CHF) and diabetes mellitus (DM) are associated with INR instability. Valve replacement (VR) with mechanical valve is an independent predictor of elevated warfarin sensitivity index. Left atrium (LA) and Glutamic pyruvic transaminase were tested in [26], whose effect cannot be entirely certain. They need further testing.

**Table 1:** clinic variables and genotypes used for warafrin dose prediction in this study

	variables
information	Age, Height, Weight, Gender
genotypes	CYP2C9, VKORC1
drug and habit	Amiodarone, Drinking
clinical variables	LA (Left atrium), ALT (Glutamic pyruvic transaminase), SCr (Serum creatinine), CHR (Congestive Heart Failure), DM (Diabetes Mellitus), VR(valve Replacement)
INR	target INR

### Data imbalance and minority class

In the dataset of warfarin patients, the unequal number of patients in clinical or genotype variables, which have a large influence on warfarin dose-effect, lead to data imbalance. For instance, both CYP2C9 and VKORC1 account for a large proportion of dosage variability. Patients with rare genotypes of CYP2C9 and VKORC1 sometimes require an unusually large or small stable dosage. Meanwhile, patients who take amiodarone or ingest alcohol, probably need a reduction on the daily dosage. We partitioned minority and majority based on “genotype”, “amiodarone” and “drinking”, and observed the data imbalance in terms of three clinical variables.

**Fig. 1:**
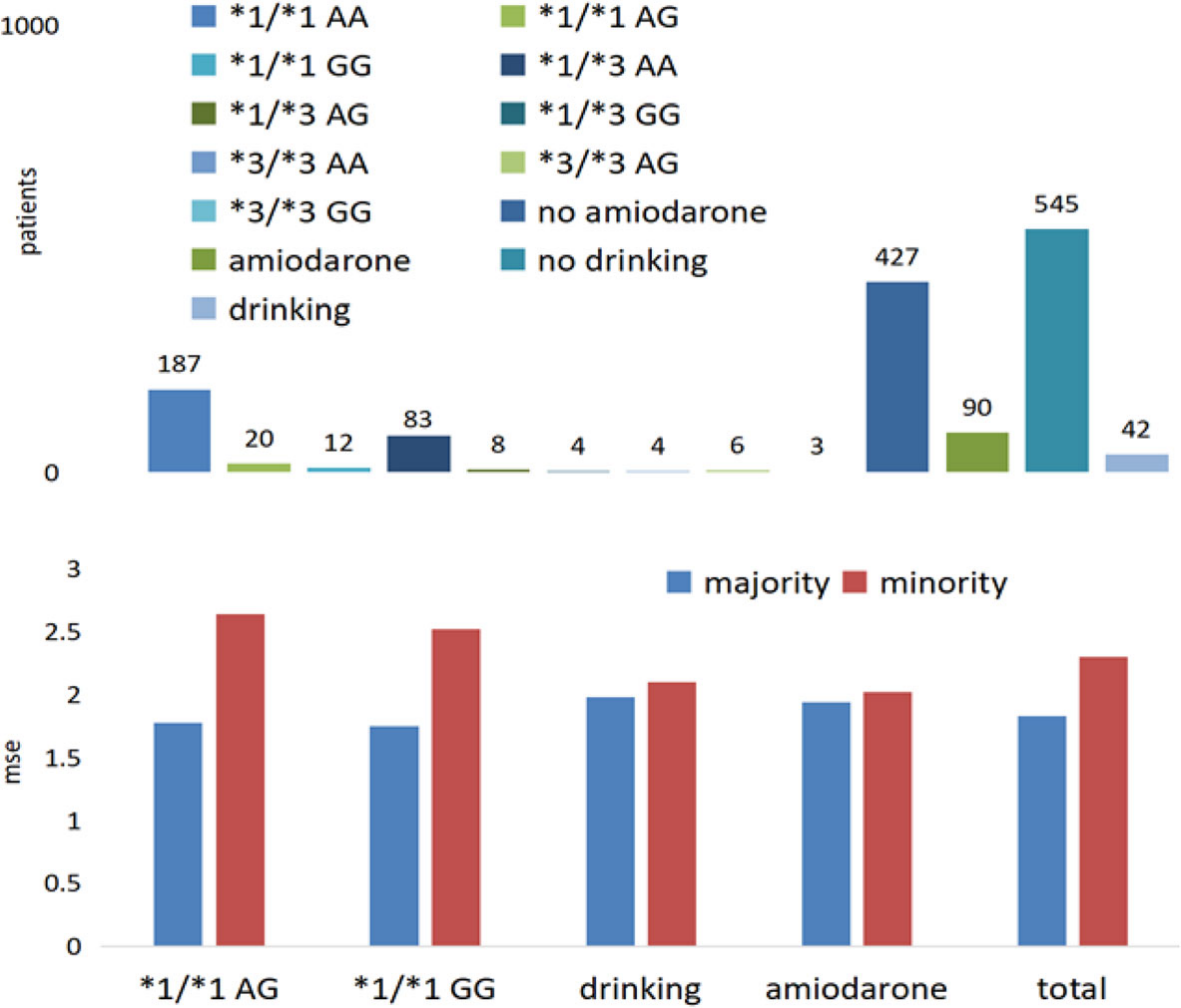
minority groups and the effect of minority on predictive accuracy

The data imbalance can be observed from Fig. 1. The patients who have rare genotypes are the minority and ones who have popular genotypes (i.e. *1/*1 for CYP2C9, AA for VKORC1) are the majority. The proportion of minority and majority in a dataset is about 1:13, may be even smaller. Furthermore, patients with *3/*3 for CYP2C9 and AG for VKORC1 are even fewer. Besides, the patients, who took amiodarone or drank regularly, are grouped as the minority and the other ones can be the majority. The number of patients, who took amiodarone, is 90, only account for 15.33%; the number of drinking patients is 42 and account for 7.15%. Some samples may be duplicated in groups of minorities when samples are partitioned based on different clinical variables. In this case, the duplicated samples only remain in the group of minorities, which has fewer samples.

## Results

### Experiment

In the experiment, we take RF, BRT, SVR and Warfarindosing as the baselines in warfarin dose prediction. ESMOTE with BRT/RF and SMOTE with RF are also compared with our method. The results and comparison were discussed in Section “Discussion”.

The initial setting on the parameters of DBCSMOTE and RF/BRT are given in Table 2. For DBSCAN and SMOTE, the parameters are the number of minimum samples in a neighbourhood centered on a core-sample, the radius and the oversampling ratio combination, which were denoted as MinPts, Eps (i.e. *ε*) and {*r*}, respectively. #*iteration* indicates the number of iterations of the greedy strategy in DBCSMOTE. A RF is composed of 100 classification and regression trees (CARTs), while BRT is composed of 150 trees*.* For trees in RF, the minimum number of leaves of a parent node is 12 (the depth of tree is small). In BRT, the number of leaves of a weak tree model is 12. It is noted that only the initial range of some parameters are given in Table 2, not the optimized value of parameters. DBCSMOTE-RF/BRT and all the comparison methods run on the machine with CPU of 3.1GHz speed and cache of 4G.

**Table 2:** setting of experimental parameters

DBCSMOTE-BRT/RF	Parameter	Value
DBSCAN	MinPts	{2,3,4,5}
	Eps or *ε*	[0.95,1.2]
SMOTE	*r*	[0.2, 1.5]
greedy strategy	*#iteration*	100
BRT	*#max_leaves*	12
	*#tree*	150
	*#features*	15
RF	*min_leaves*	12
	*#tree*	100
	*#features*	15
	*tree type*	CART

In this study, the collected dataset was sampled five times in sequence to generate five training, validation and test sets, respectively. We guarantee each sample in the dataset will be used for training and test. This is to evaluate the overall performance of models on different data distributions. DBCSMOTE-BRT/RF and all the comparison methods will run ten times on each training set, and for each training set, the best trained predictive model was used to calculate the average value of evaluation metrics in figures.

### Evaluation metrics

We use four metrics to evaluate the performance of models in warfarind dose prediction. They are *R*-squared (*R*^2^), mean square error (*mse*), mean absolute error (*mae*) and a probability bias (20%-*p*). The forms of *mse, mae* and *R*^2^ are denoted as (1).

1 $$ {\displaystyle \begin{array}{l} mse=\frac{1}{N}{\sum}_{i=1}^N{\left({y}_i-{\hat{y}}_i\right)}^2\\ {} mae=\frac{1}{N}{\sum}_{i=1}^N\mid {y}_i-{\hat{y}}_i\mid \\ {}{R}^2=1-\frac{\sum_{i=1}^N{\left({y}_i-{\hat{y}}_i\right)}^2}{\sum_{i=1}^N{\left({y}_i-{\overline{y}}_i\right)}^2}\end{array}} $$

Here, *N* indicates the number of samples, *ŷ*_*i*_ and *y*_*i*_ are predicted and observed speed, respectively. A small *mse* indicates small variance of errors, a small *mae* indicates small deviation of model and a large *R*^2^ leads to good fitness of model.

All the methods use the same number of variables to build the predictive model. We give the alternative models a fair chance, which means using same times of running and on the same hardware.

### Effect of greedy strategy

**Fig. 2:**
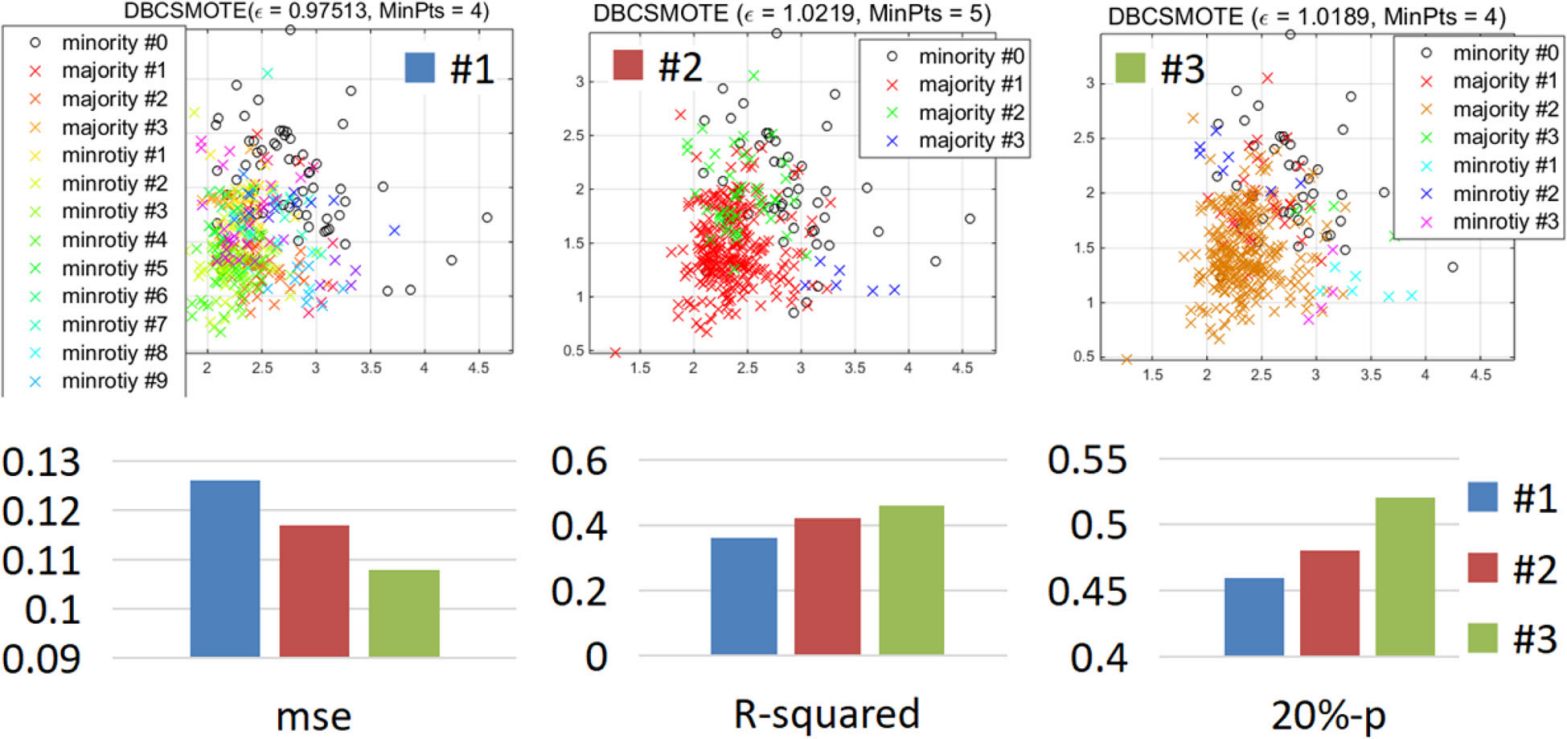
*mse*, *R*-squared and 20%-*p* of different parameters for DBCSMOTE

In this study, a greedy strategy was used to optimize the parameters of DBCSMOTE (Table 2). We select three combinations of MinPts, *ε* and {*r*}, denoted as #1, #2 and #3, which were obtained by the greedy strategy, to analyze the effect of parameter optimization. The average *mse*, *R*^2^ and 20%-*p* of #1, #2 and #3 can be observed in Fig. 2. It can be seen that DBCSMOTE #1, *ε* of 0.97, MinPts of 4 and *r* of {0.2,1.9,0.3,1.3,0.2...}, generated the most clusters (i.e. 16) but got the largest error and the smallest *R*^2^ in dose prediction. DBCSMOTE #2 created the smallest number of clusters, which yielded higher accuracy than #1 but lower accuracy than #3. DBCSMOTE #3 generated four minority clusters and yielded the best performance in dose prediction, i.e. *mse* of 1.08, *R*^2^ of 0.48 and 20%-*p* of 52%. It was observed that when *ε* is set to an appropriate value, the number of clusters will be reasonable and minority samples would be more effective.

The results in Fig. 2 illustrate the necessity of the greedy strategy used in DBCSMOTE. The parameters of DBCSMOTE have a large influence on the predictive accuracy. Greedy strategy can search a group of good parameters for DBCSMOTE with a small computational effort, which is much lower than that of ESMOTE. DBCSMOTE can achieve a good trade-off between predictive accuracy and computational effort by using greedy search.

### Predictive accuracy

**Fig. 3:**
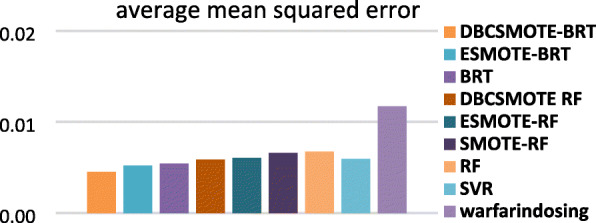
average *mse* of methods over five test sets

**Fig. 4:**
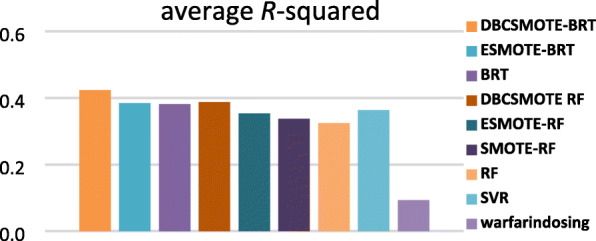
average *R*^2^ of methods over five test sets

**Fig. 5:**
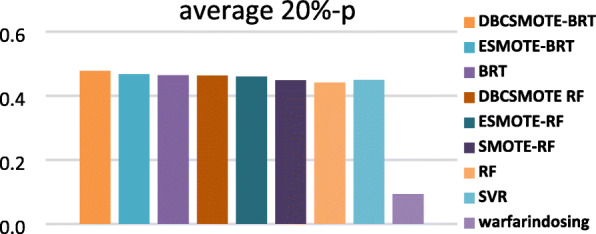
average 20%-*p* of methods

In this subsection, we compared DBCSMOTE-BRT/RF with the other models to observe the performance of different modeling techniques in warfarin dose prediction. Figures 3, 4 and 5 show the comparison results in terms of four evaluation metrics on five test sets. Small *mse*, large *R*^2^ and 20%-*p* of a predictive model implies high accuracy, a highly fitting quality and a good clinical application of the model.

It was observed in Figs. 3, 4 and 5 that DBCSMOTE-BRT got an average *R*^2^ of 0.424, average *mse* of 0.013 and average 20%-*p* of 47.8%, which performed the best among these models. As compared to ESMOTE-BRT, DBCSMOTE-BRT outperformed 25.7% in *R*^2^ and got a 4.9% reduction on *mse*. As compared to the base predictive model BRT, DBCSMOTE-BRT increased by 25.8% in *R*^2^ and got an overall 6.3% reduction on *mse*. Meanwhile, DBCSMOTE-BRT yielded better results than RF-series models. It illustrated that DBCSMOTE was surprisingly suitable for BRT in warfarin dose prediction. It was also noted that DBCSMOTE-RF outperformed ESMOTE-RF and SMOTE-RF in these cases, although it performed worse than DBCSMOTE-BRT.

As shown in Figs.3 and 4, the average *mse* and *R*^2^ of DBCSMOTE-BRT/RF were generally smaller and higher than that of ESMOTE-BRT, ESMOTE-RF and SMOTE-RF, respectively. It demonstrated that both BRT and RF combined with DBCSMOTE can be trained on synthetic samples of higher quality than that combined with ESMOTE and SMOTE in these cases.

We also observed the performance of baselines such as SVR, BRT, RF and Warfarindosing. Among these baselines, SVR got the similar performance as ESMOTE-RF, but was worse than BRT series models. Warfarindosing is a tool built by a traditional regression method, which is the worst one among these models. It is concluded that the base predictive models such as BRT and RF combined with oversampling technique can outperform baselines in warfarin dose prediction.

In terms of 20%-*p*, it was noted in Fig. 5 that the comparison methods achieved the value larger than 45%, except Warfarindosing. Among these methods, DBCSMOTE-BRT obtained the largest 20%-*p* of 47.8%. Due to the conflict between *mse* and 20%-*p* in some cases, a small *mse* may not lead to a large 20%-*p*. Fortunately, DBCSMOTE-BRT obtained a not bad 20%-*p* in the case that it has got a small *mse*.

## Discussion

### Advantage and limitation of DBCSMOTE

ESMOTE-BRT/RF and SMOTE-RF were similar models as DBCSMOTE-BRT/RF, which were trained on the training set extended by adding new synthesized samples. Since the base predictive models that DBCSMOTE, SMOTE and ESMOTE combined with are same, the better generalization and predictive accuracy of DBCSMOTE-BRT/RF can be attributed to the new samples synthesized by DBCSMOTE.

**Fig. 6:**
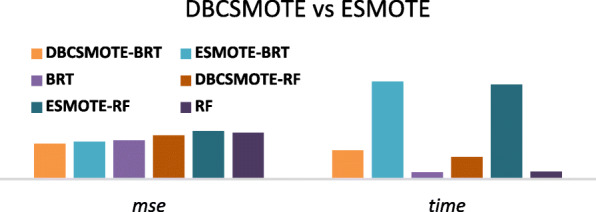
DBCSMOTE vs ESMOTE in *mse* and computational time

Meanwhile, DBCSMOTE has a better trade-off between the computational time and the quality of minority clusters than that of ESMOTE. For example, as in Fig. 6, on a single training set, DBSMOTE can outperform ESMOTE 5.2 ~ 8.3% in *mse* reduction with paying a much shorter computational time (i.e. average 87 s of one run). ESMOTE usually spent a long evolutionary time on calculating the fitness of oversampling parameters even it can find high-quality oversampling parameters. Moreover, the minority groups used for ESMOTE were generated based on the human knowledge, which cannot be generalized to different data distributions. While the minority groups of DBCSMOTE were generated according to data distribution. Therefore, DBCSMOTE was obviously a cost effective oversampling method and can be more widely used in different data distribution of test sets.

The generalization is a double-edged sword for DBCSMOTE. On different datasets, DBCSMOTE can performed generally well, but cannot always be the best solution. On four out of five test sets, ESMOTE can lead to equally good or better predictive model than DBCSMOTE. This is attributed to ESMOTE’s better oversampling ratio combinations and the high-quality synthesized samples. In some cases, the greedy strategy in DBCSMOTE cannot perform as well as ESMOTE in parameters optimization. For ESMOTE, the cost of a long optimization time sometimes receive rich returns. Another limitation of DBCSMOTE is that the predictive accuracy of DBCSMOTE model strongly depends on the quality of minority clustering. Once the clustered minority samples are not accurate, the predictive accuracy cannot be improved anyway.

### Effect of LA, ALT and SCr

**Fig. 7:**
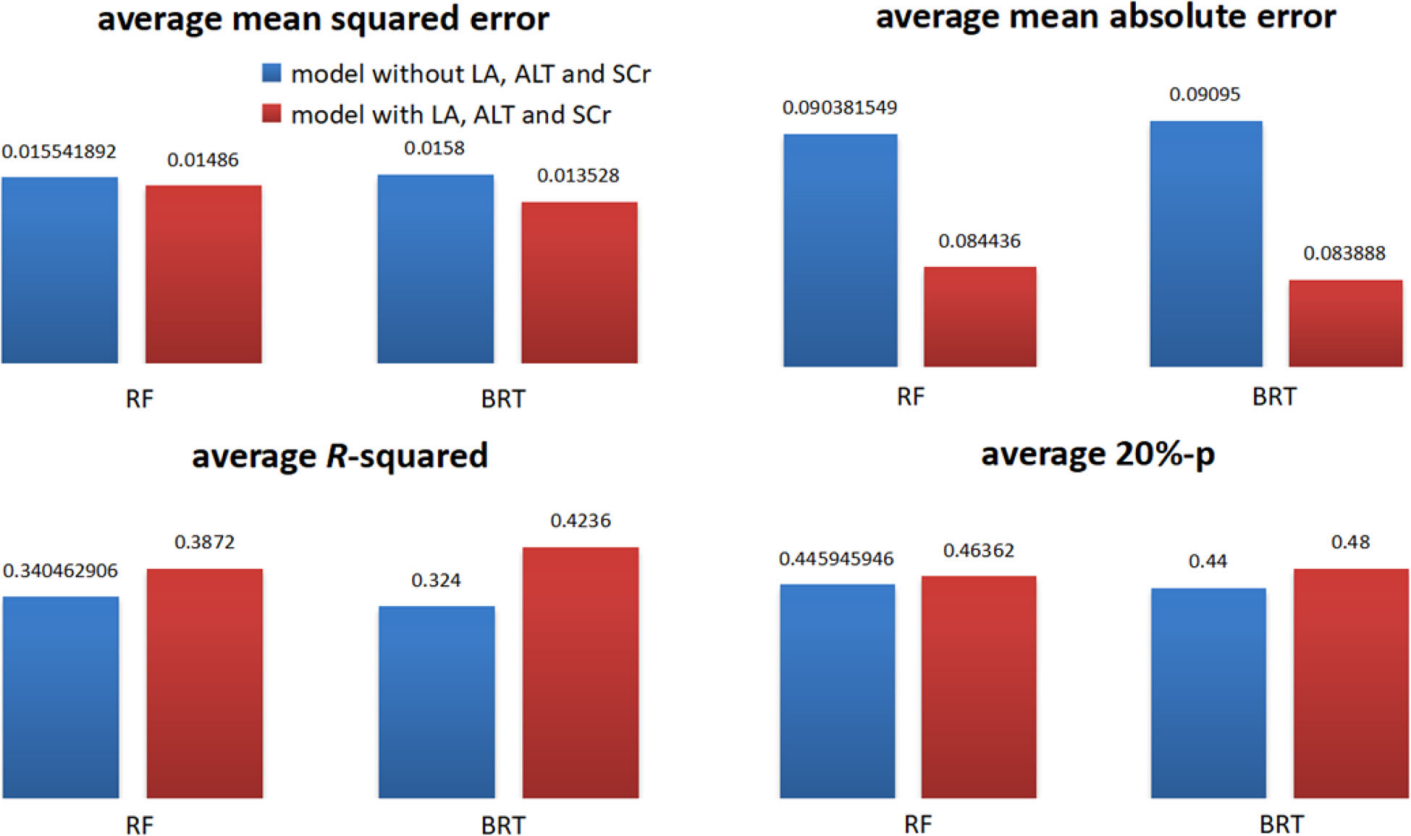
comparison between DBCSMOTE-BRT/RF models with and without LA, ALT and SCr (**a**) *mse* comparison (**b**) *mae* comparison (**c**) *R*^2^ comparison (**d**) 20%-*p* comparison

In the feature group shown in Table 1, the clinical variables, amiodarone and drinking, have been tested and proven to be effective for dose prediction in previous papers [26, 27]. As reported in [26, 27], ALT and SCr didn’t significantly reduce the predictive error in some cases. This conclusion was based on the small accuracy reduction of their ensemble model without using ALT and SCr. However, the trees in the ensemble model, which were randomly generated by a genetic programming on a small dataset of 289 samples, may haven’t made full use of ALT and SCr. Hence, the inference that LA, ALT and SCr have no significant effect on dosage was probably not accurate. In this study, we test the effect of LA, ALT and SCr in DBCSMOTE-BRT and -RF on the larger dataset of 592 samples for warfarin dose prediction. The comparison results are shown in Fig. 7. The average *mse*, *mae*, *R*^2^ and 20%-*p* for comparison models were also calculated on five test sets.

The value of ALT indicates the pathological changes of liver, where produces clotting factors. If the liver is damaged, clotting factors will be reduced and the body’s clotting mechanism will not work. SCr is the indicator of renal function. According to the research work [28], acute renal injury (AKI) can be caused by volume hemorrhage caused by excessive anticoagulation. Traditionally, warfarin alone was not the cause of renal injury. However, this report of warfarin induced AKI have changed this view, a phenomenon known as warfarin related nephropathy (WRN). Therefore, the patients of AKI probably should reduce the warfarin dosage for achieving the target INR. According to the guideline [29], left atrium (LA) diameter > 50 mm was a patient-related risk factor for thrombosis, which probably has an influence on the warfarin daily dosage.

It is noted in Fig. 7 that by adding LA, ALT and SCr to the predictive model, both DBCSMOTE-BRT and -RF obtained a smaller *mse/mae,* larger *R*^2^ and 20%-*p*. By using LA, ALT and SCr, the improvement of models on *R*^2^ was up to 13.7% and the reduction on *mse/mae* was 4.38%/6.57%. This illustrates that LA, ALT and SCr have a certain influence on warfarin dose-effect.

### Effect of CHR, DM and VR

**Fig. 8:**
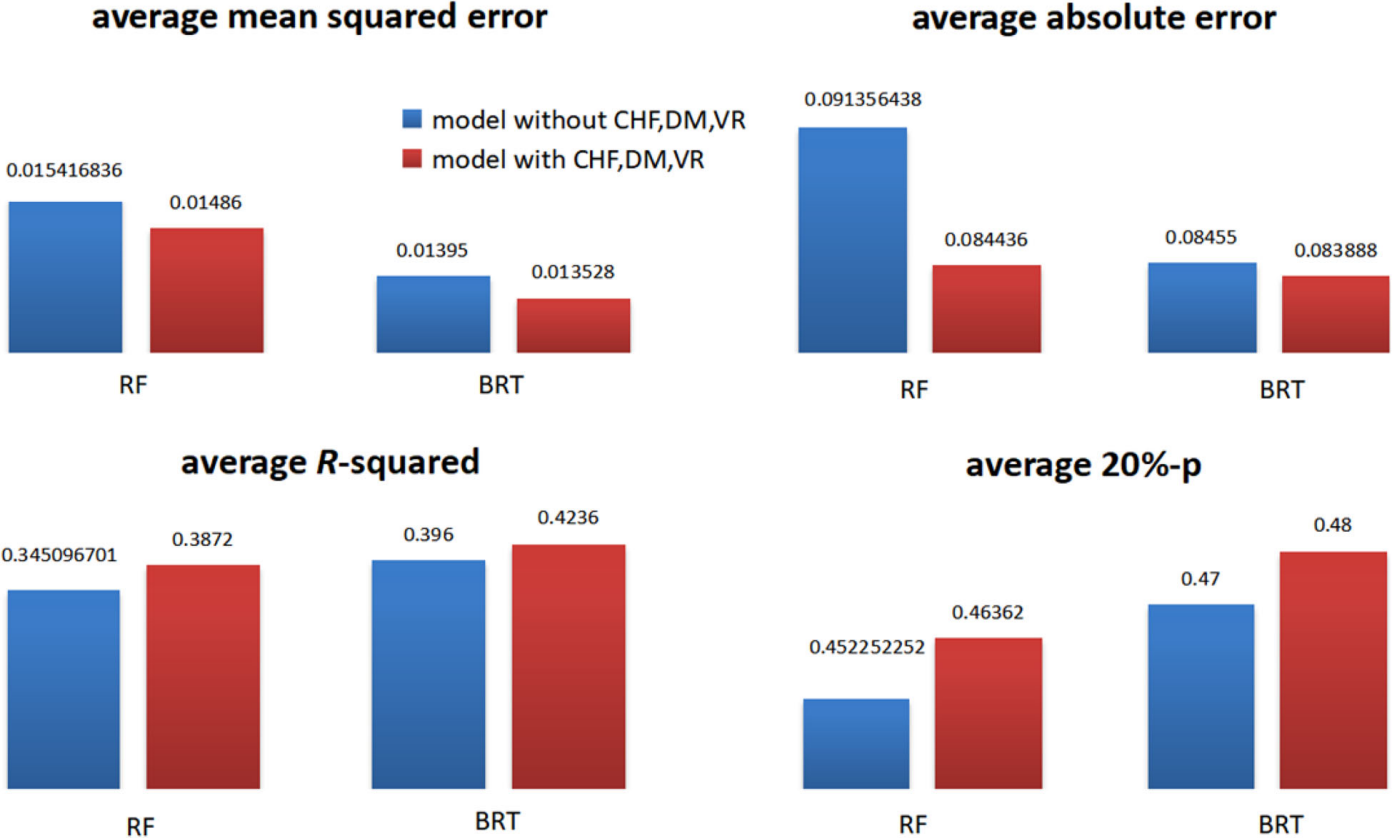
comparison between DBCSMOTE-BRT/RF models with and without CHF, DM and VR (**a**) *mse* comparison (**b**) *mae* comparison (**c**) *R*^2^ comparison (**d**) 20%-*p* comparison

In this subsection, we further test the effect of CHR, DM and VR in DBCSMOTE-BRT/RF for warfarin dose prediction. The evaluations for models are the same as that in the subsection, which tested LA, ALT and SCr. The comparison results of models are shown in Fig. 8.

The patients, undergoing either mechanical heart valve or Bioprosthetic valve replacement (VR), need anticoagulation therapy. However, the dose-effect of VR for warfarin was not clear. Recently, more and more diabetics, especially older patients, need anticoagulation therapy due to cardiovascular and cerebrovascular complications of diabetes mellitus (DM). Congestive Heart Failure (CHF) is an uncertainty factor for warfarin treatment. It was reported that CHF was related to the occurrence rate of deep vein thrombosis in Japan [30]. This relationship hasn’t been proven for Chinese patients.

Observed from Fig. 8, DBCSMOTE-BRT/RF using CHR, DM and VR obviously outperformed the ones without CHR, DM and VR. For DBCSMOTE-RF, there was a large improvement when using CHR, DM and VR, i.e. about 3.6% reduction on *mse*, 7.5% reduction on *mae*, a 12.2% increase on *R*^2^ and 2.5% increase on 20%-*p*. For DBCSMOTE-BRT, when using CHR, DM and VR, it generally obtained a 3.0% reduction on *mse*, 6.8% reduction on *mae*, a 7.9% increase on *R*^2^ and 2.1% increase on 20%-*p*. Meanwhile, it was discovered that the model with CHR, DM and VR didn’t always work better than the model without CHR, DM and VR. On two of five test sets, the model without CHR, DM and VR obtained a smaller error and a larger *R*-squared. This illustrates that CHR, DM and VR probably have a certain impact on warfarin dose-effect for a special part of patients, maybe not so effective for the other patients.

### Clinical application

In this study, DBCSMOTE was still tested on a small dataset. It is known that a large dataset may have substantially different statistical characteristics between balanced and unbalanced samples, which a small dataset may not have. However, this may be not an issue to DBCSMOTE. DBCSMOTE literately conducts the clustering on minority and majority classes by the feedback of predicted dosage during training. It can be automatically adaptive to the characteristics between balanced and unbalanced samples in a new dataset, maybe a large one.

According to the performance of variables in prediction, the features that contain genotypes and amiodarone are effective in clinical application. Meanwhile, the frequency of occurrence of DM and drinking is much smaller than that of the other variables in multiple trees. Therefore, we can conclude that Age, Height, Weight, ATL, LA, CRH, VR, amiodarone, TargetINR, CYP2C9 and VKORC1 may be effective in warfarin dose prediction. When new clinical variables (race or newly found gene variations) should be added, our model can be updated as the process implemented in the Section “METHODS”. A clustering and training process with new clinical variables was necessary. For example, the combination of the original and new variables will be used as a feature vector input. DBSCAN and SMOTE are to find minority class and create synthetic samples with new variables.

### Comparison between predictive models

Many clinical trials suggested that the advantages of pharmacogenetic-guided dosing algorithms over standard dosing are obvious, whereas the advantages over clinical variables-guided algorithm are equivocal. Different genotype-guided dosing models were utilized in these trials, such as IWPC model, Warfarindosing and Yu model. Among the three models, Warfarindosing was proven the best one [26]. It was trained on a dataset of 1015 patients and obtained a *R*^2^ = 0.21 on our test sets. IWPC model got the smallest *R*^2^ of 0.112 despite it was trained on the 4000-size dataset. Both IWPC model and Warfarindosing are recommended by a clinical guideline [3], while Yu model built on a similar dataset with ours for Chinese patients. As previous works reported [21, 22, 26], these regression models are not accurate in warfarin dose prediction.

As stated in our previous works, deep learning methods or neural networks are usually too complex for warfarin dose prediction. Three baselines, SVR, BRT and RF, have been tested in warfarin dose prediction. SVR usually can obtain a good *R*^2^, which was better than SMOTE-RF and performed as well as ESMOTE-RF in some cases. RF and BRT are two ensemble models, where BRT is more suitable for oversampling methods. This is because BRT gradually fits all the samples by adding trees to reduce the bias, making full uses of the synthesized samples of oversampling, while RF would not specifically reduce the bias to the synthesized samples of oversampling. The oversampling method makes the imbalance dataset more balance and improves the generalization of the predictive model. Apparently, two models, DBCSMOTE-BRT and ESMOTE-BRT, have potential in clinical application in the future.

## Conclusion

To solve the data imbalance problem in warfarin dose prediction, we propose a clustering-based oversampling method called DBCSMOTE. It targets to improve the balance in warfarin dosage dataset and predictive accuracy. DBCSMOTE-BRT/RF was tested on the dataset of our hospital and IWPC.

DBCSMOTE-BRT obtained a *R*^2^ of 0.478 and *mse* of 1.08. In terms of 20%-*p*, DBCSMOTE-BRT also achieved a large value of 48%. As compared to ESMOTE models, DBCSMOTE takes only a small computational time to achieve the same or higher performance in many cases. The oversampling ratio and number of minority clusters have a large impact on the effect of oversampling. According to our test, the predictive accuracy was higher when the number of minority clusters was 6 ~ 8. The oversampling ratio for the minority clusters of small size should be large (> 1.2) and that for the minority clusters of relatively large size should be small (< 0.2).

Two genotypes, CYP2C9 and VKORC1, no doubt contributed largely to the predictive accuracy of the predictive model. Moreover, three features, LA, ALT and SCr included in the model, actually improved the predictive accuracy on our test sets. When CHR, DM and VR were absent in our model, the predictive accuracy of models, BRT and RF, decreased. This illustrates the effect of these clinical features on warfarin daily dose. However, we still cannot fully illustrate the deep mechanic behind these effects of features with machine learning methods.

Future work will focus on three aspects. One is to integrate evolutionary approach with clustering on minority groups. The performance of integrated methods will be observed. The other is to develop dose refining algorithm, which makes uses of INR value on days of therapy so as to improve the accuracy of dose prediction. The final work is to interpret deep mechanic of the clinical features used for warfarin dose prediction.

## Methods

**Fig. 9:**
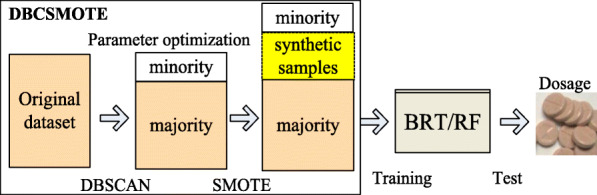
DBCSMOTE extending training set, parameter optimization of DBCSMOTE and testing process

We propose a density-based clustering synthetic minority oversampling technique (DBCSMOTE) to accomplish warfarin dose prediction under the data imbalance. DBCSMOTE contains a density-based clustering algorithm and an oversampling method, together with a parameter optimizer. The flow of DBCSMOTE in extending a training set, parameter optimization and algorithm testing are illustrated in Fig. 9. BRT and RF are the base predictive models combined with DBCSMOTE for warfarin dose prediction.

### Density-based clustering for the minority class

Density-based clustering algorithm groups together samples with many nearby neighbours, marking as outliers samples that lie alone in low-density regions, whose nearest neighbours are too far away. The density-based spatial clustering of application with noise (DBSCAN), one of the most common density-based clustering algorithms, is robust to outliers. In this study, we make use of the robustness of DBSCAN to outliers and clusters to find the minority groups. DBSCAN has two parameters, one is the radius, denoted as Eps or *ε*, which represents the area of circular neighbourhood centred on a given sample *p,* and the other is the number of minimum samples in a neighbourhood centred on the sample *p*, denoted as MinPts.

A sample *p* is a core-sample if at least MinPts samples are within distance *ε* of it, including *p*; a sample *p* is a boundary-sample if less than MinPts samples are within *ε* of it, but it is within distance *ε* from one core-sample. These samples compose clusters and the left ones are outliers. To compose minority group, the minority sample *q* is defined as follow:

- *q* is an outlier not reachable from any other sample;
- *q* belongs to the cluster, which has a smaller number of members than a threshold (i.e. 50 in this study).

**Fig. 10:**
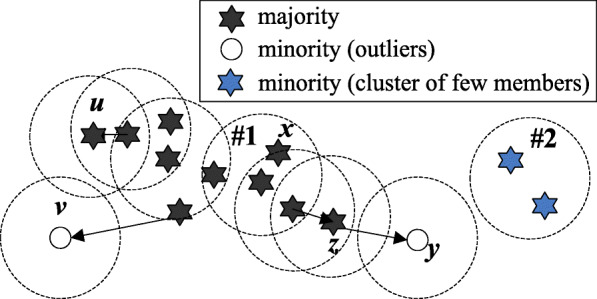
finding samples of the minority class according to the density

Figure 10 shows a diagram of density-based clustering on samples. Let us describe the process as follow: if MinPts = 4, ten black points and two blue points form two clusters, denoted as #1 and #2. The left points, *v* and *y*, are two outliers*.* According to our definition, the samples of cluster #1 are majority members. In this case, two minority groups are created. One group consists of two blue points (cluster #2), which are fewer than 3 (threshold); the other group is composed of two outliers.

If Eps is too large, too many samples will be clustered in the same cluster. If Eps is too small, a cluster will split. When MinPts becomes large, many samples may be clustered as outliers. A too-small MinPts will lead to a large number of majority samples. Conventionally, it is appropriate to use *k*-distance graph to obtain the value of Eps. Euclidean distance is employed to calculate the distance between samples. The valley point position in *k*-distance curve is a good value of Eps if using descending order. In this study, only a small number of samples with special features are exactly needed. We hope these samples can be clustered as outliers. Hence, Eps can be a little larger than that at valley point position.

### Synthetic samples by SMOTE

The task of synthesizing new samples is accomplished by SMOTE. Synthetic samples are generated with two steps: selecting a minority sample and finding *k*-nearest neighbours of the minority sample. Let *x* be the minority sample. *k*-nearest neighbours are the ones, whose distance to *x* is *k* nearest. The distance is evaluated as (2).

2 $$ l={\left\Vert {wx}_i-{wx}_j\right\Vert}^2 $$

Where *x*_*i*_ and *x*_*j*_ are two samples. *w* is the weight vector, which is assigned to variables of a sample. Some variables such as “drinking”, “amiodarone” and “genotypes” deserve large weight. All the variables are normalized.

Let *n* be one of *k* nearest neighbours to *x* and *δ* be the random value in [0,1]. A new sample *x’* is as the form.

3 $$ x^{\prime }=x+\delta \cdot \left(x-n\right) $$

Among the variables, someones are 0/1 logical variables, which are set to binary value (0/1). Hence, rounding resets the value of these variables. For a minority sample, *k*-nearest neighbours are selected from both the majority and the minority class.

Let *r* be the oversampling ratio of DBCSMOTE. The sampling number can be denoted as *N*_*s*_ = round(r*N_*mino*_), where *N*_*mino*_ is the number of samples in the minority class. If *N*_*s*_ < *N*_*mino*_ (*r* < 1), randomly select *N*_*s*_ minority samples from *N*_*mino*_ and create each synthetic sample by randomly selecting one of its nearest neighbour; else if *N*_*s*_ > *N*_*mino*_ (*r >* 1), let *φ* = (int)*N*_*s*_/*N*_*mino*_ and *ρ =* [*r*-(int)*N*_*s*_/*N*_*mino*_] < 1, create φ*N_*mino*_ synthetic samples by using *φ* nearest neighbours of each minority sample, and φ*N_*mino*_ synthetic samples are generated as the way under *N*_*s*_ < *N*_*mino*_ (*r* < 1).

The dosage *f* of new samples is the weighted dosage sum of neighbours, which is denoted as the form.

4 $$ f\left(x^{\prime}\right)=\alpha \cdot f\left({n}_j\right)+\left(1-\alpha \right)\cdot f\left(\hat{x}\right) $$

Here, *α* = 1/*l*. The weight of dosage is calculated by the distance reciprocal of a new sample to its parent.

### Greedy strategy for parameters optimization

It is hard for a human to configure the parameters for DBCSMOTE, that will make the accuracy of the predictive model highest. As for DBSCAN, two parameters MinPts and Eps need to be optimized. For SMOTE, the oversampling ratio combination, denoted as {*r*}, needs to be optimized.

The optimization problem is to find the best parameters of DBCSMOTE (i.e. DBSCAN and SMOTE), which lead to the smallest error of the predictive models such as BRT and RF. Let *X* = [MinPts, Eps, {*r*}] be the variable of objective function *z*(*X*). The optimization problem can be formulated as follow.

5 $$ \min z(X)=\mathrm{K}\cdot \frac{2.5\cdot {z}_1(X)+{\mathrm{z}}_2(X)}{z_3(X)} $$

Where,

$$ {\displaystyle \begin{array}{l}{z}_1(X)={mse}_{train}+2\cdot {mse}_{valid}\\ {}{z}_2(X)={mse}_{train}\left(f>4.2|f<1.5\right)\\ {}{z}_3(X)={R^2}_{train}+{R^2}_{valid}\end{array}} $$

The objective function *z*(*X*) contains three sub functions. z_1_(*X*) calculates the mean squared error of *X* with respect to the training and validation sets, respectively. z_2_(*X*) calculates the mean squared error with respect to the samples, whose observed dosage *f* is larger than 4.2 mg/day or smaller than 1.5 mg/day. z_3_(*X*) calculates the *R*^2^ of *X* on the training and validation sets, respectively. K is an amplification constant in *z*(*X*) only for observing *z*(*X*). We set K to 20, which makes *z*(*X*) > 1.

In (5), *mse*_*train*_ and *mse*_*valid*_ indicate the *mse* on the training and validation sets, respectively. *R*^2^_*train*_ and *R*^2^_*valid*_ indicate the values of *R*^2^ on the training and validation sets, respectively. For *mse* and *R*^2^ calculation, a temporal predictive model-CART, which is built on the training set extended by *X*, is used. This is a low-complexity model with convenient use for evaluation on parameters of DBCSMOTE in each iteration.

A greedy strategy is used to evaluate the quality of DBCSMOTE and search the solution to the optimization problem (5). The flow of the greedy strategy is as follow.

- Step 1: Give a set of feasible solutions {*X*} (feasible setting of parameters);
- Step 2: Select a candidate *X* in {*X*};
- Step 3: Run DBCSMOTE with *X* to generate an extended training set;
- Step 4: Create a CART model on the extended training set and validated on the validation set;
- Step 5: Calculate the *mse* and *R*^2^ of the CART model, and then calculate the value of *z*(*X*);
- Step 6: Traverse all candidates in {*X*}; the candidate that obtained the smallest value of *z*(*X*) represents the best solution (parameters) of DBCSMOTE.

### DBCSMOTE for dose prediction

In previous works [21, 22], a predictive software called “WarfarinSeer v1.0” was developed by our research group, which has encapsulated some predictive models such as SMOTE-RF, CART, RF, BRT, SVR and Ensemble models [26]. This predictive software is written in MATLAB and can successfully run with the environmental support of MATLAB. In order to provide clinicians with a new model for warfarin dose prediction, we developed an upgraded version “WarfarinSeer v2.0” based on DBCSMOTE-BRT/RF models.

The flow of DBCSMOTE-BRT/RF is as follow:

Step 1: Divide the original dataset into three subsets, i.e. training set, validation set and test set;

Step 2: Run the greedy strategy to optimize the parameters of DBCSMOTE;

Step 3: Keep minority and majority clusters with these optimized parameters;

Step 4: Use SMOTE to synthesize new samples for extending the training set;

Step 5: Build the BRT/RF model on the extended training set, validate it on the validation set, and output dose prediction on the test set.

**Fig. 11:**
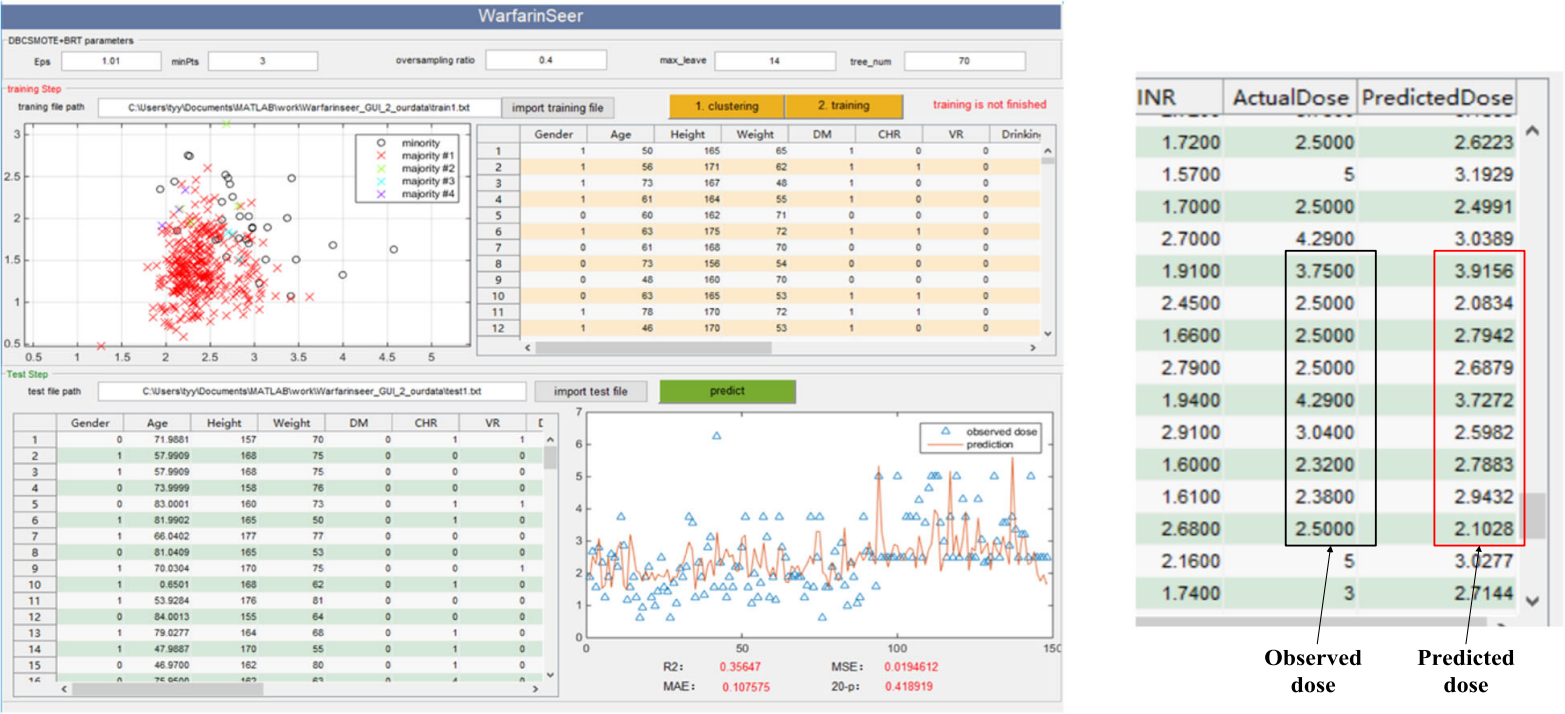
warfarin dosing predictive tool “WarfarinSeer” encapsulated DBCSMOTE (**a**) software interface (**b**) actual dose vs predicted dose

Figure 11 shows the GUI of “WarfarinSeer v2.0”. On the GUI, data grids present the training data and test data, which are imported from an outside file with suffix “.dat”. In the file, each row indicates one sample (i.e. a patient). The columns indicate clinical features and the last column is the stable dosage. Text-boxes receive the parameter configuration for algorithms and different models. Button “1. clustering” is to run DBCSMOTE with greedy strategy optimization. Button “2. training” starts the training of BRT predictive model on the extended training set yielded by DBCSMOTE, and button “predict” runs the trained BRT model to predict test samples. Charting controls display the process of model fitting and results of running algorithms. The performance of the predictive model, mean square error, *R*-squared and 20%-*p*, will be displayed at right-bottom.

## Supplementary information

**Additional file 1.** DBCSMOTE.zip, code files for generating minority and majority clusters in Matlab. DBCSMOTE_demo.m: the demo of DBCSMOTE together with random forest, which gives the estimated dosage. ‘num’ indicates the number of iterations of running DBCSMOTE. In each iteration, ‘evaluatePop’ calls the function to evaluate the oversampling quality. ‘train.txt’, ‘validate.txt’ and ‘test.txt’ are sub sets used for training, validation and testing. DBSCAN_fun.m: the function of algorithm DBSCAN. It conducts the clustering with two parameters (Eps and MinPts) on an input dataset and returns the samples of minority clusters and the number of clusters. RandomForest.m: the function of random forest. Random forest is an ensemble model of CARTs, which are the weak regression models. They are built on the extended training set, which is extended by DBCSMOTE. CARTprediction.m: the function of CART algorithm. This is a weak regression model of random forest. Meanwhile, this is the tool for evaluating the oversampling quality, which is generated by DBCSMOTE.

## Data Availability

The datasets collected from the First Affiliated Hospital of Soochow University and analyzed during the current study are not publicly available. This is because the warfarin data are private information of the First Affiliated Hospital of Soochow University, and cannot be publicly without the permission of the Hospital, but are available from the corresponding author on reasonable request (zhangyuzhen@suda.edu.cn).

## Abbreviations

IWPC: International Warfarin Pharmacogenetics Consortium
SMOTE: Synthetic minority oversampling technique
ESMOTE: Evolutionary synthetic minority oversampling technique
BRT: Boosted regression tree
RF: Random forest
CART: Classification and regression tree
ANN: Artificial neural network
SVR: Support vector regression
DBSCAN: Density-based spatial clustering of application with noise
*mse*: mean squared error
*mae*: mean absolute error
AKI: Acute renal injury
